# Supplementary figures and images for: Dynamics of a Protein Interaction Network Associated to the Aggregation of polyQ-Expanded Ataxin-1
Source: Genes (Basel). 2020 Sep 25;11(10):1129. doi: 10.3390/genes11101129 (PMC7600199; doi:10.3390/genes11101129)

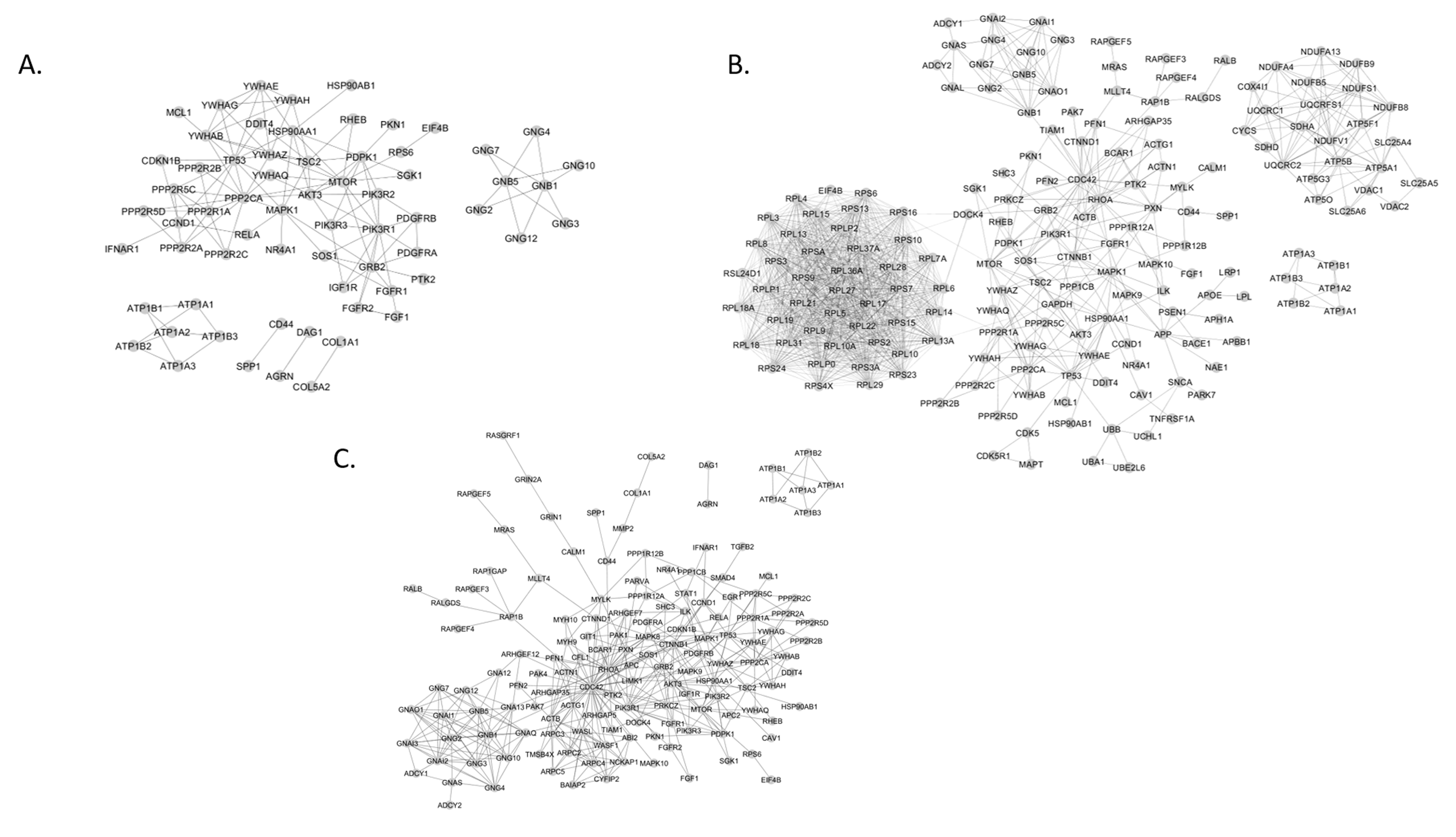

Supplement: Supplementary file 1 [file genes-11-01129-s001.zip › Supplementary Material/Supplementary Figure 1 new.tif]

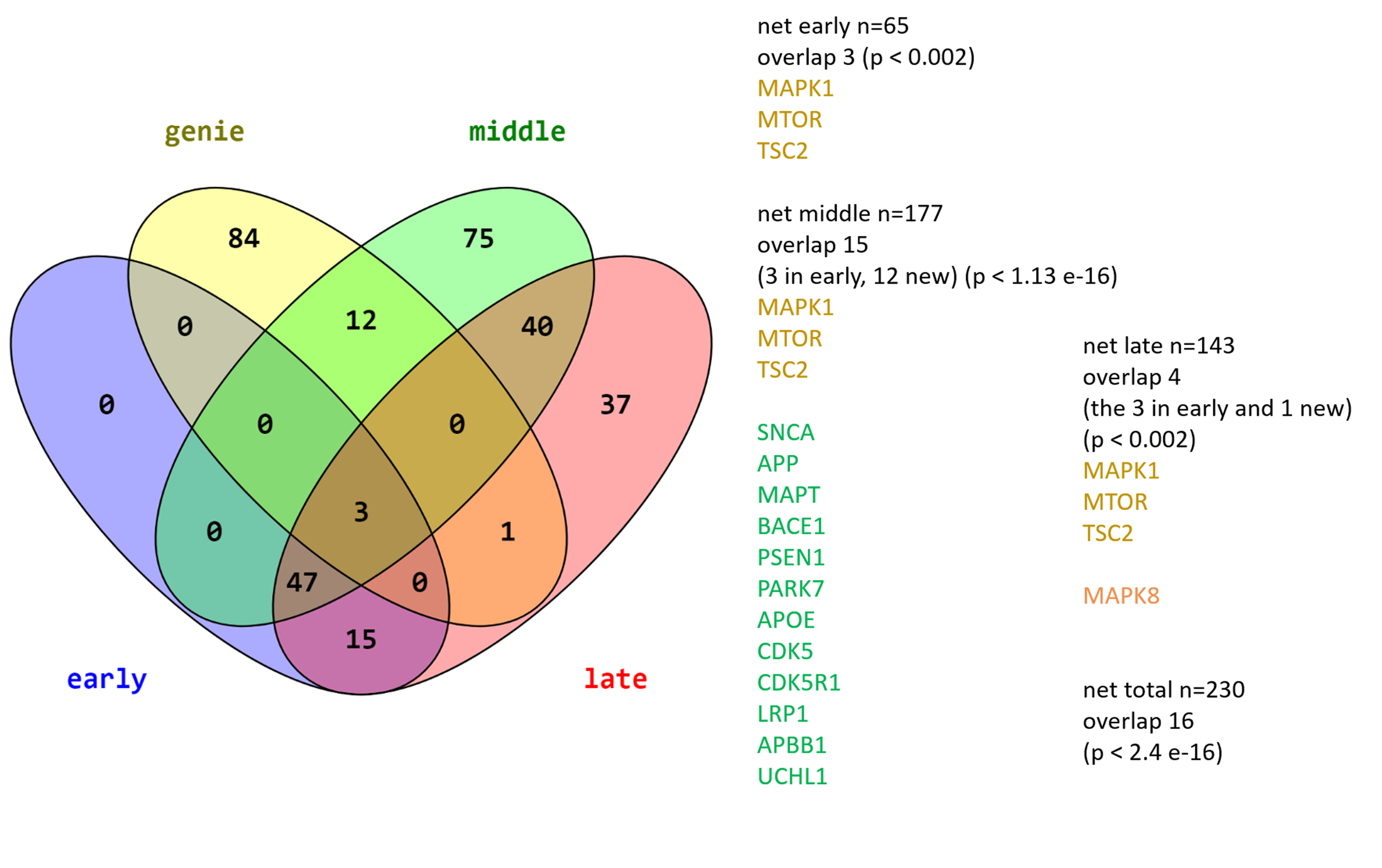

Supplement: Supplementary file 1 [file genes-11-01129-s001.zip › Supplementary Material/Supplementary Figure 2.tif]

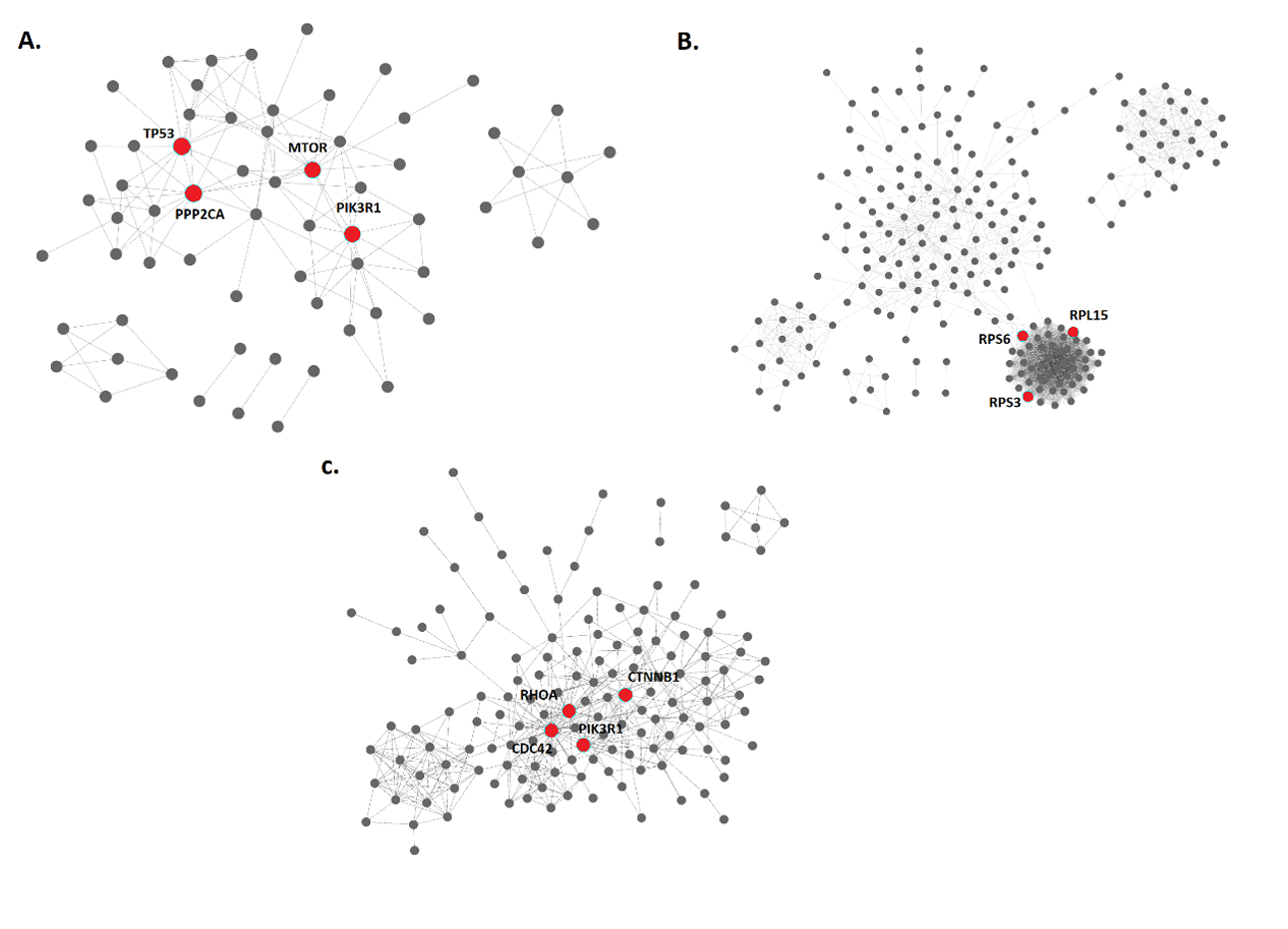

Supplement: Supplementary file 1 [file genes-11-01129-s001.zip › Supplementary Material/Supplementary Figure 3.tif]

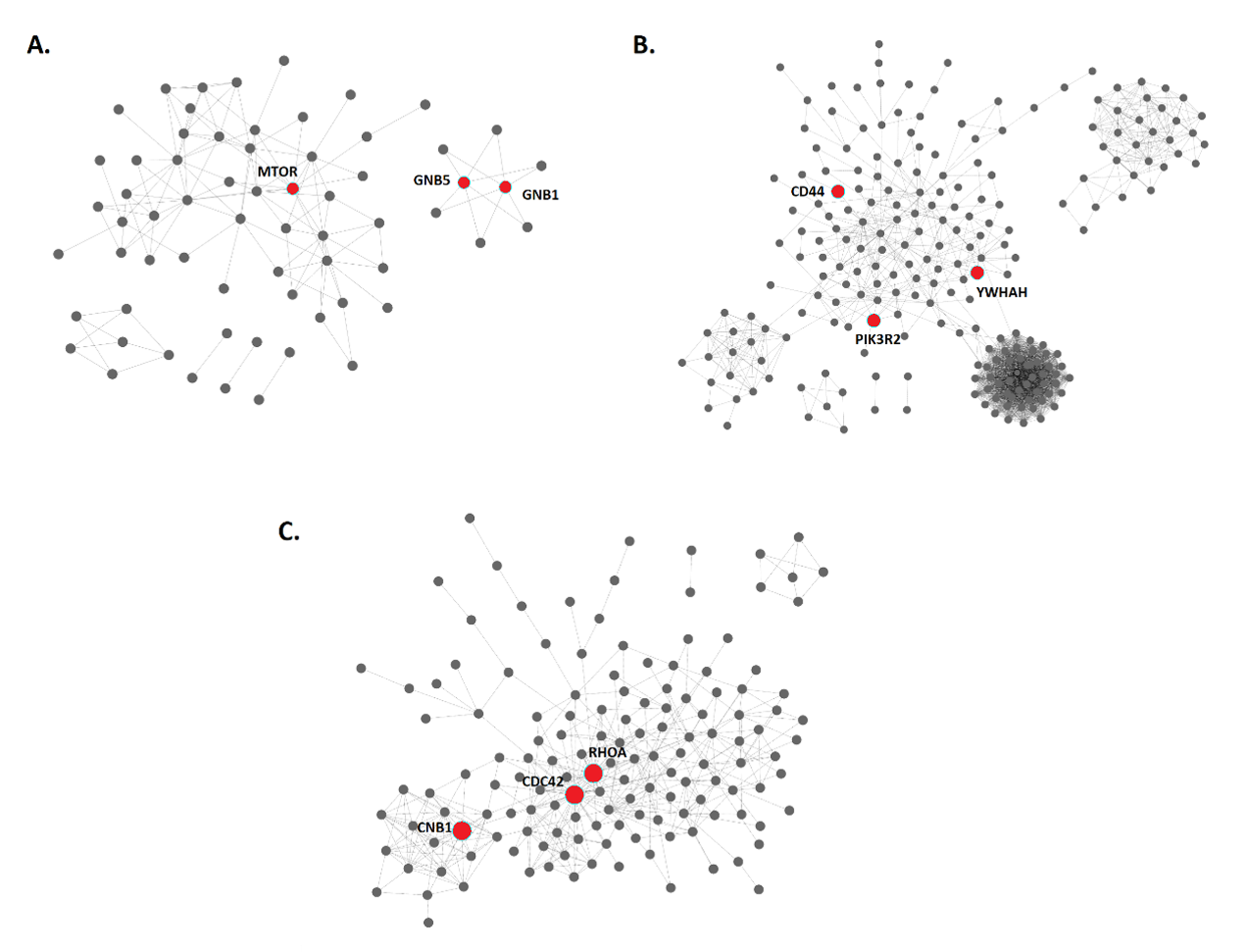

Supplement: Supplementary file 1 [file genes-11-01129-s001.zip › Supplementary Material/Supplementary Figure 4.tif]

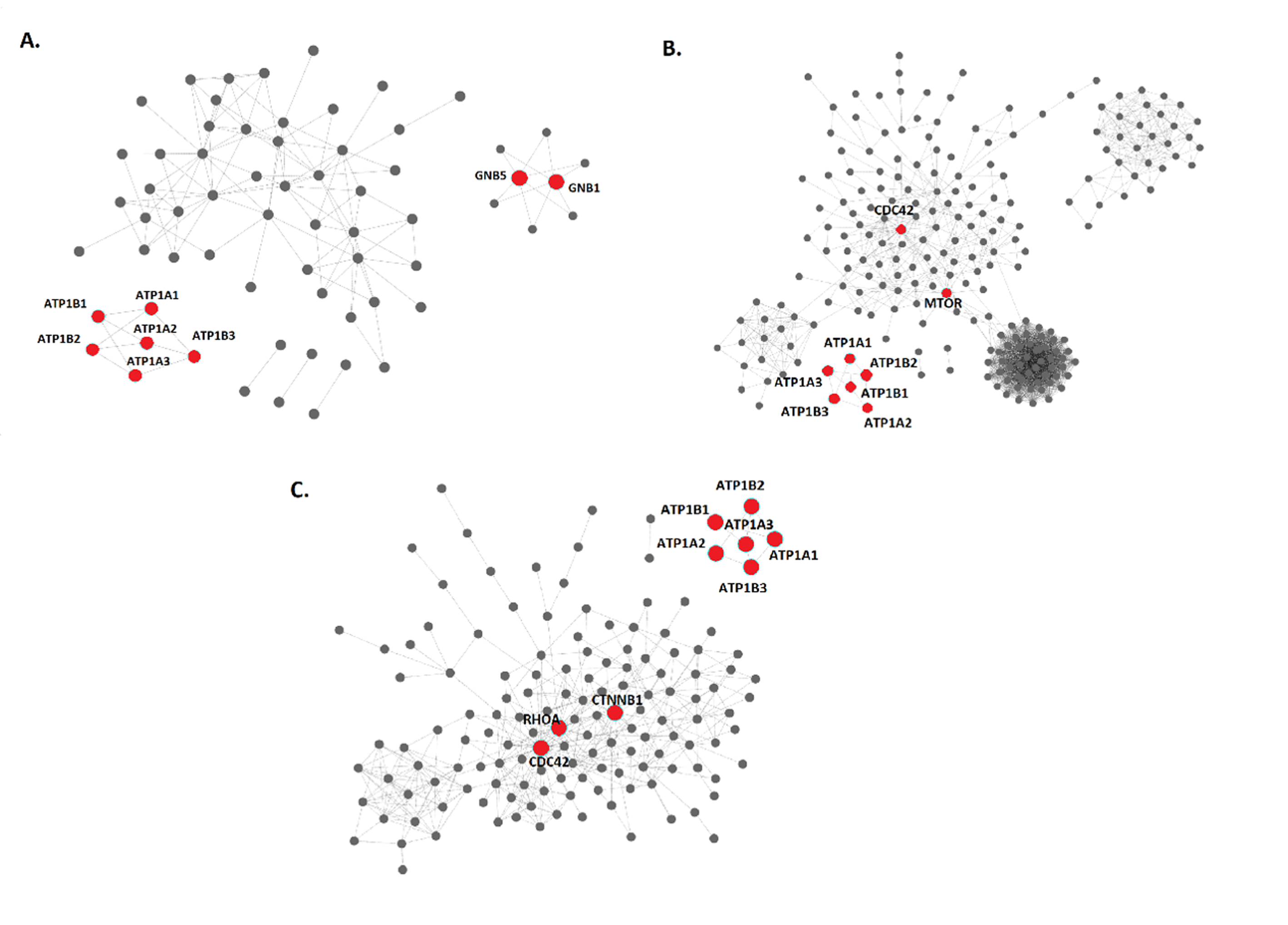

Supplement: Supplementary file 1 [file genes-11-01129-s001.zip › Supplementary Material/Supplementary Figure 5.tif]

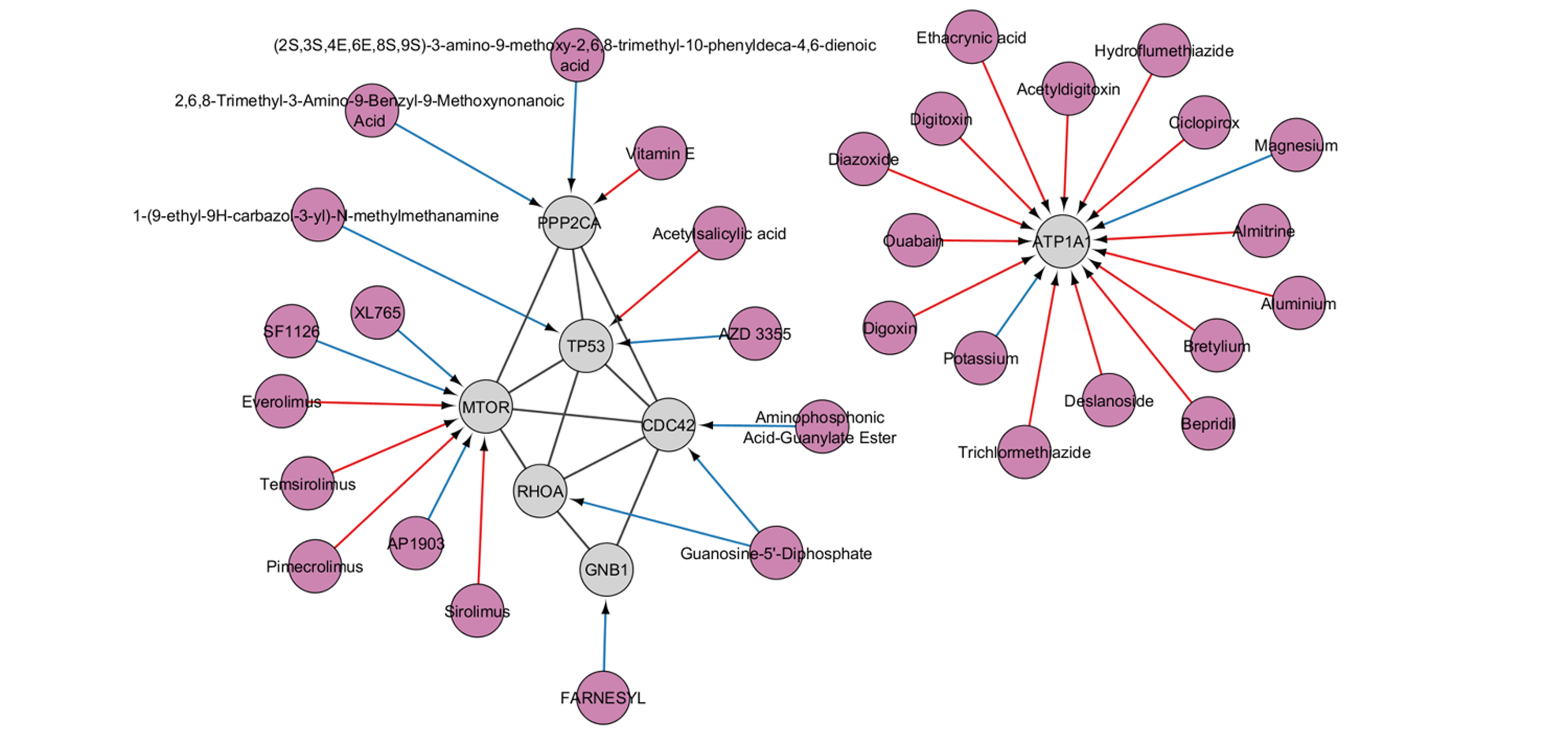

Supplement: Supplementary file 1 [file genes-11-01129-s001.zip › Supplementary Material/Supplementary Figure 6.tif]
